# Supplementary material for: Recipient Pericardial Apolipoprotein Levels Might Be an Indicator of Worse Outcomes after Orthotopic Heart Transplantation
Source: Int J Mol Sci. 2024 Feb 1;25(3):1752. doi: 10.3390/ijms25031752 (PMC10855207; doi:10.3390/ijms25031752)
Supplement: Supplementary file 1 [file ijms-25-01752-s001.zip › ijms-2803795-supplementary.pdf]

SUPPLEMENTARY TABLES

Figure S1. Correlations with preoperative laboratory parameters

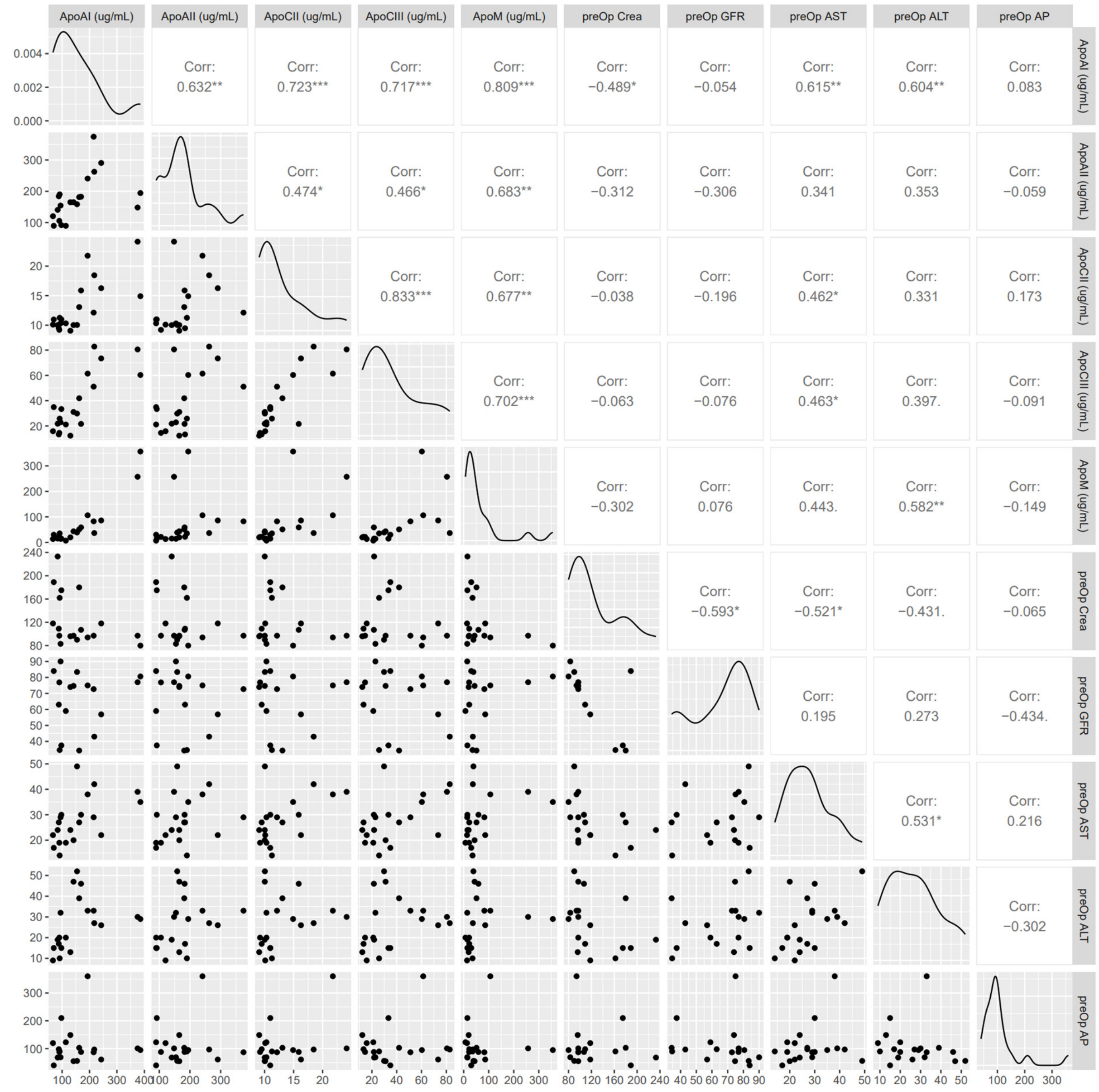

AOPP: advanced oxidation protein products; ALP: alkaline phosphatase; ALT: alanine transaminase, Apo: apolipoprotein; AST: aspartate aminotransferase; GGT: gamma-glutamyl transpeptidase; IL: interleukin; LDH: lactate dehydrogenase; T3: triiodothyronine; T4: thyroxine; TNF: tumor necrosis factor

Figure S2. Correlations with postoperative laboratory parameters

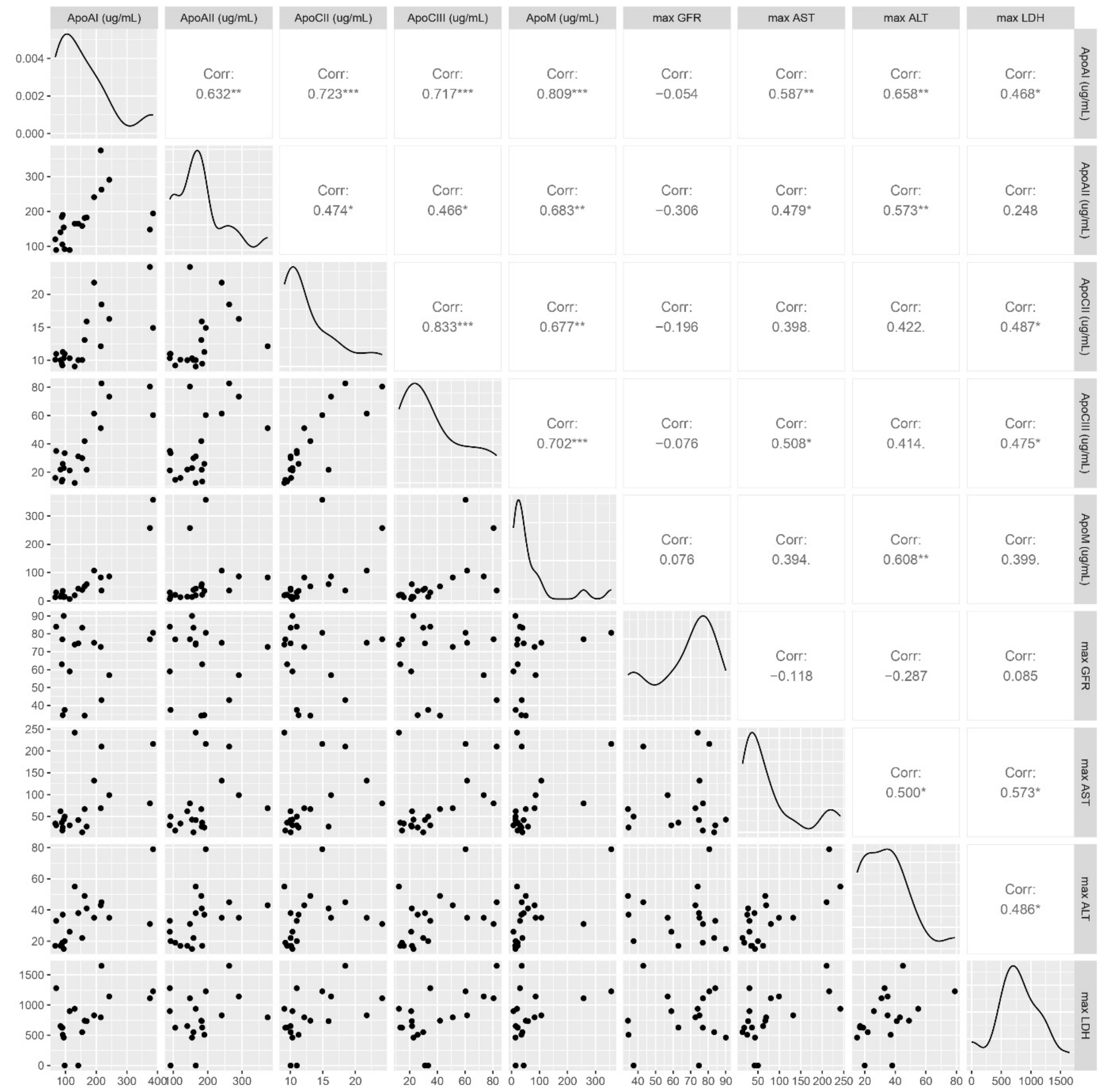

AOPP: advanced oxidation protein products; ALP: alkaline phosphatase; ALT: alanine transaminase, Apo: apolipoprotein; AST: aspartate aminotransferase; GGT: gamma-glutamyl transpeptidase; IL: interleukin; LDH: lactate dehydrogenase; T3: triiodothyronine; T4: thyroxine; TNF: tumor necrosis factor

Table S1. Relationship between recipient interleukin levels and primary graft dysfunction

	No PGD		PGD		p value
	Median	IQR (25-75)	Median	IQR (25-75)	
thiol (414 nm)	0.00	0.00-2.05	1.58	0.00-5.27	0.385
thiol (405 nm)	0.00	0.00-1.93	0.75	0.0-5.28	0.682
IFNg (pg/mL)	2.28	1.50-2.78	5.35	1.17-8.14	0.211
IL-17A (pg/mL)	8.50	8.42-8.60	8.97	8.44-9.33	0.178
IL-17F (pg/mL)	17.23	16.99-17.90	20.72	17.27-24.28	0.211
IL-21 (pg/mL)	30.13	18.39-43.88	31.91	20.83-50.30	0.750
IL-22 (pg/mL)	17.11	17.09-17.13	17.13	17.09-17.22	0.494
IL-4 (pg/mL)	21.59	17.25-25.71	45.89	23.54-61.73	0.064
TNF (pg/mL)	3.18	3.06-3.35	3.67	3.09-4.00	0.211
IL-10 (pg/mL)	19.30	6.10-19.30	12.70	6.10-49.00	0.963
IL-13 (pg/mL)	5.72	5.63-5.99	7.28	5.7-9.34	0.211
IL-2 (pg/mL)	20.13	15.11-27.82	49.57	16.78-77.33	0.178
IL-5 (pg/mL)	0.98	0.88-1.12	1.65	0.86-1.86	0.178
IL-6 (pg/mL)	200.06	27.29-580.64	251.41	46.89-705.01	0.892
IL-9 (pg/mL)	17.55	16.74-19.88	18.23	17.17-21.71	0.554
Leptin (ng/mL)	7.54	6.71-10.44	6.36	5.55-6.62	0.029
Adiponectin (ng/mL)	37.44	26.85-47.34	20.58	17.49-46.90	0.335
Adipsin (ng/mL)	683.52	597.48-783.62	657.55	585.08-739.55	0.750
oxLDL (ng/mL)	461.39	339.93-687.64	304.20	232.75-761.47	0.437
ApoAI (ug/mL)	127.57	90.12-187.11	172.59	84.68-216.75	0.820
ApoAII (ug/mL)	161.99	125.58-188.69	213.85	108.73-346.45	0.494
ApoB100 (ug/mL)	328.82	226.70-570.65	307.38	258.85-762.86	0.963
ApoCII (ug/mL)	10.65	10.03-15.64	11.55	9.55-16.87	1.000
ApoCIII (ug/mL)	27.82	21.28-55.72	43.00	17.96-74.85	0.554
ApoD (ug/mL)	13.30	8.92-22.51	16.47	10.85-25.31	0.617
ApoE4 (ug/mL)	0.00	0.00-0.00	0.00	0.00-0.00	0.750
ApoH (ug/mL)	0.00	0.00-8.42	2.48	0.00-8.99	0.820
ApoJ (ug/mL)	0.00	0.00-0.00	0.00	0.00-22.59	0.892
ApoM (ug/mL)	37.14	14.81-79.29	33.42	22.39-71.05	1.000
AOPP	0.00	0.00-0.70	0.00	0.00-0.00	0.494
T3 (pg/mL)	3.48	2.83-4.01	3.53	2.37-3.97	0.892
T4 (ug/dL)	4.62	4.18-5.31	4.42	3.61-5.70	0.617

AOPP: advanced oxidation protein products; Apo: apolipoprotein; IFNg: interferon-γ; IL: interleukin; IQR: interquartile range; PGD: primary graft dysfunction; oxLDL: oxidized low-density lipoprotein; T3: triiodothyronine; T4: thyroxine; TNF: tumor necrosis factor

Table S2. Relationship between recipient interleukin levels and postoperative mechanical circulatory support

	No MCS		MCS		p value
	Median	IQR (25-75)	Median	IQR (25-75)	
thiol (414 nm)	0.00	0.00-2.10	0.00	0.00-4.57	0.612
thiol (405 nm)	0.00	0.00-2.08	0.00	0.00-4.02	1.000
IFNg (pg/mL)	2.35	1.46-2.86	3.79	1.25-7.73	0.306
IL-17A (pg/mL)	8.50	8.42-8.60	8.86	8.37-9.25	0.349
IL-17F (pg/mL)	17.25	16.99-17.96	20.20	16.64-23.27	0.445
IL-21 (pg/mL)	29.62	18.24-39.98	35.75	23.25-50.17	0.445
IL-22 (pg/mL)	17.11	17.11-17.13	17.11	17.08-17.20	1.000
IL-4 (pg/mL)	22.45	17.25-25.93	39.81	16.81-58.48	0.395
TNF (pg/mL)	3.18	3.05-3.36	3.37	3.11-3.99	0.230
IL-10 (pg/mL)	19.30	6.10-19.30	19.30	6.10-39.10	0.933
IL-13 (pg/mL)	5.72	5.64-5.99	7.04	5.43-8.73	0.553
IL-2 (pg/mL)	20.13	14.78-29.50	38.86	14.78-71.65	0.266
IL-5 (pg/mL)	0.98	0.87-1.12	1.45	0.79-1.86	0.349
IL-6 (pg/mL)	182.30	26.47-612.01	390.38	68.74-648.22	0.612
IL-9 (pg/mL)	17.54	16.72-20.10	19.11	17.24-20.89	0.349
Leptin (ng/mL)	7.61	6.70-10.50	6.53	5.77-6.81	0.042
Adiponectin (ng/mL)	37.04	26.17-45.03	21.29	18.29-99.93	0.933
Adipsin (ng/mL)	705.01	607.55-788.67	640.79	580.32-717.80	0.445
oxLDL (ng/mL)	451.86	337.54-594.76	347.07	242.28-875.79	0.933
ApoAI (ug/mL)	113.50	89.79-169.01	193.14	99.83-216.18	0.553
ApoAII (ug/mL)	158.66	120.53-184.14	240.82	127.55-318.48	0.230
ApoB100 (ug/mL)	327.02	216.72-451.73	350.28	260.73-1065.77	0.395
ApoCII (ug/mL)	10.32	10.03-14.91	12.14	10.02-20.00	0.497
ApoCIII (ug/mL)	25.82	21.17-41.92	51.08	23.61-72.09	0.266
ApoD (ug/mL)	12.02	8.86-21.84	19.31	11.78-25.80	0.306
ApoE4 (ug/mL)	0.00	0.00-0.00	0.00	0.00-0.00	0.672
ApoH (ug/mL)	0.00	0.00-10.66	1.70	0.00-7.64	0.612
ApoJ (ug/mL)	0.00	0.00-0.00	0.00	0.00-15.06	1.000
ApoM (ug/mL)	35.39	14.73-58.89	36.66	24.98-94.47	0.553
AOPP	0.00	0.00-0.00	0.00	0.00-3.21	0.933
T3 (pg/mL)	3.66	2.83-4.05	3.28	2.65-3.93	0.800
T4 (ug/dL)	4.68	4.23-5.37	4.23	3.59-5.34	0.230

AOPP: advanced oxidation protein products; Apo: apolipoprotein; IFNg: interferon-γ; IL: interleukin; IQR: interquartile range; MCS: mechanical circulatory support; oxLDL: oxidized low-density lipoprotein; T3: triiodothyronine; T4: thyroxine; TNF: tumor necrosis factor

Table S3. Relationship between recipient interleukin levels and vasoplegia

	No Vasoplegia		Vasoplegia		p value
	Median	IQR (25-75)	Median	IQR (25-75)	
thiol (414 nm)	0.00	0.00-2.15	0.94	0.00-4.95	0.617
thiol (405 nm)	0.00	0.00-1.93	0.74	0.00-5.28	0.750
IFNg (pg/mL)	2.39	1.64-3.71	1.53	0.59-3.17	0.211
IL-17A (pg/mL)	8.54	8.45-8.81	8.42	8.33-8.60	0.178
IL-17F (pg/mL)	17.57	17.02-19.75	17.01	16.44-17.91	0.178
IL-21 (pg/mL)	30.13	18.52-46.94	29.64	20.70-32.87	0.750
IL-22 (pg/mL)	17.11	17.11-17.13	17.08	17.08-17.15	0.211
IL-4 (pg/mL)	23.32	17.25-38.08	25.59	18.11-38.08	1.000
TNF (pg/mL)	3.25	3.12-3.37	3.10	3.00-3.36	0.211
IL-10 (pg/mL)	19.30	6.10-19.3	12.70	6.10-29.20	0.820
IL-13 (pg/mL)	5.94	5.64-6.89	5.65	5.36-6.15	0.249
IL-2 (pg/mL)	22.14	17.12-36.86	15.45	10.76-32.17	0.211
IL-5 (pg/mL)	1.03	0.93-1.39	0.87	0.71-1.15	0.148
IL-6 (pg/mL)	225.56	26.22-684.09	165.13	47.96-262.20	0.682
IL-9 (pg/mL)	17.91	16.84-20.33	17.33	16.82-17.56	0.385
Leptin (ng/mL)	6.85	5.98-8.97	8.98	6.76-11.21	0.335
Adiponectin (ng/mL)	33.93	19.86-53.61	31.61	22.51-41.53	0.750
Adipsin (ng/mL)	654.83	597.48.1-758.71	757.50	581.52-921.98	0.437
oxLDL (ng/mL)	447.10	339.93-694.79	361.36	173.21-663.83	0.385
ApoAI (ug/mL)	113.75	88.43-187.11	178.34	120.53-235.64	0.290
ApoAII (ug/mL)	161.92	125.58-188.69	227.97	108.71-353.48	0.437
ApoB100 (ug/mL)	324.93	256.72-441.92	593.94	209.66-890.51	0.682
ApoCII (ug/mL)	10.98	10.03-15.64	11.23	10.10-15.23	0.892
ApoCIII (ug/mL)	27.82	17.29-55.72	41.07	23.64-67.84	0.554
ApoD (ug/mL)	12.34	8.92-20.34	28.69	15.85-37.66	0.022
ApoE4 (ug/mL)	0.00	0.00-0.00	0.00	0.00-0.00	0.750
ApoH (ug/mL)	0.00	0.00-4.14	5.17	0.00-10.58	0.682
ApoJ (ug/mL)	0.00	0.00-0.00	3.44	0.00-24.31	0.385
ApoM (ug/mL)	32.78	16.23-56.95	62.10	16.08-85.20	0.682
AOPP	0.00	0.00-0.07	0.00	0.00-0.00	0.494
T3 (pg/mL)	3.67	2.83-4.04	3.15	1.57-3.57	0.211
T4 (ug/dL)	4.46	4.08-4.88	5.52	4.45-6.02	0.178

AOPP: advanced oxidation protein products; Apo: apolipoprotein; IFNg: interferon-γ; IL: interleukin; IQR: interquartile range; oxLDL: oxidized low-density lipoprotein; T3: triiodothyronine; T4: thyroxine; TNF: tumor necrosis factor

Table S4. Relationship between recipient interleukin levels and mortality

	No Mortality		Mortality		p value
	Median	IQR (25-75)	Median	IQR (25-75)	
thiol (414 nm)	0.00	0.00-2.10	0.00	0.00-4.57	0.612
thiol (405 nm)	0.00	0.00-2.08	0.00	0.00-4.02	1.000
IFNg (pg/mL)	2.21	1.46-2.86	3.47	1.32-5.35	0.445
IL-17A (pg/mL)	8.49	8.42-8.66	8.57	8.44-8.97	0.445
IL-17F (pg/mL)	17.21	16.99-18.18	17.96	16.95-20.72	0.445
IL-21 (pg/mL)	30.65	18.83-45.18	28.08	17.77-41.764	0.672
IL-22 (pg/mL)	17.11	17.11-17.13	17.11	17.08-17.13	0.349
IL-4 (pg/mL)	20.72	17.25-32.87	24.19	20.28-45.89	0.395
TNF (pg/mL)	3.18	3.05-3.37	3.36	3.09-3.69	0.497
IL-10 (pg/mL)	19.30	6.10-19.30	6.10	6.10-25.90	0.497
IL-13 (pg/mL)	5.72	5.62-6.30	5.99	5.62-7.28	0.445
IL-2 (pg/mL)	20.13	14.78-29.50	30.83	16.11-49.57	0.306
IL-5 (pg/mL)	0.98	0.87-1.23	1.12	0.89-1.66	0.306
IL-6 (pg/mL)	217.83	29.76-612.01	112.44	22.11-549.25	0.553
IL-9 (pg/mL)	17.57	16.72-20.41	17.36	16.96-19.61	1.000
Leptin (ng/mL)	7.61	5.90-10.50	6.65	6.36-6.86	0.230
Adiponectin (ng/mL)	30.00	19.87-43.02	45.03	18.99-74.48	0.553
Adipsin (ng/mL)	705.01	594.12-788.67	640.79	576.17-717.80	0.445
oxLDL (ng/mL)	442.34	337.54-594.76	509.02	285.15-813.87	0.553
ApoAI (ug/mL)	113.50	89.79-162.28	217.32	142.28-380.36	0.066
ApoAII (ug/mL)	165.18	120.53-184.14	194.43	119.10-318.48	0.395
ApoB100 (ug/mL)	319.23	216.72-412.51	610.30	307.38-1009.02	0.098
ApoCII (ug/mL)	10.31	10.02-13.07	14.91	11.55-21.30	0.042
ApoCIII (ug/mL)	22.84	15.84-33.39	60.32	43.00-81.66	0.005
ApoD (ug/mL)	14.57	9.50-22.74	9.93	4.88-23.31	0.349
ApoE4 (ug/mL)	0.00	0.00-0.00	0.00	0.00-405.68	0.197
ApoH (ug/mL)	0.00	0.00-0.00	10.33	2.48-36.26	0.053
ApoJ (ug/mL)	0.00	0.00-0.00	30.12	0.00-44.01	0.066
ApoM (ug/mL)	22.00	14.73-51.12	82.52	33.42-306.90	0.053
AOPP	0.00	0.00-0.00	0.00	0.00-2.28	1.00
T3 (pg/mL)	3.28	2.47-3.90	3.84	3.44-4.04	0.230
T4 (ug/dL)	4.56	2.23-5.37	4.61	3.78-5.37	0.670

AOPP: advanced oxidation protein products; Apo: apolipoprotein; IFNg: interferon-γ; IL: interleukin; IQR: interquartile range; oxLDL: oxidized low-density lipoprotein; T3: triiodothyronine; T4: thyroxine; TNF: tumor necrosis factor

Table S5. Relationship between recipient interleukin levels and rejection

	No Rejection		Rejection		p value
	Median	IQR (25-75)	Median	IQR (25-75)	
thiol (414 nm)	0.00	0.00-2.15	0.00	0.00-2.37	0.820
thiol (405 nm)	0.05	0.00-2.17	0.00	0.00-1.12	0.437
IFNg (pg/mL)	2.07	1.00-3.49	2.90	2.24-3.71	0.385
IL-17A (pg/mL)	8.50	8.41-8.65	8.62	8.48-8.81	0.335
IL-17F (pg/mL)	17.23	16.93-18.34	17.74	17.12-20.43	0.554
IL-21 (pg/mL)	30.13	18.54-45.64	27.29	17.56-42.83	0.750
IL-22 (pg/mL)	17.11	17.09-17.13	17.12	17.08-17.15	0.892
IL-4 (pg/mL)	22.45	17.46-40.03	23.32	17.25-35.91	0.750
TNF (pg/mL)	3.18	3.05-3.41	3.35	3.26-3.37	0.290
IL-10 (pg/mL)	12.70	6.10-19.30	19.30	19.30-29.20	0.211
IL-13 (pg/mL)	5.72	5.63-6.42	5.99	5.70-7.14	0.617
IL-2 (pg/mL)	20.13	13.77-35.52	26.82	20.80-36.86	0.290
IL-5 (pg/mL)	0.98	0.87-1.21	1.17	0.97-1.39	0.385
IL-6 (pg/mL)	200.06	28.08-684.09	210.07	21.81-462.51	0.682
IL-9 (pg/mL)	17.55	16.97-20.33	17.96	16.74-19.18	0.682
Leptin (ng/mL)	7.10	5.98-9.98	6.97	6.73-12.67	0.617
Adiponectin (ng/mL)	29.96	19.86-42.28	74.48	35.51-131.70	0.039
Adipsin (ng/mL)	683.52	608.82-781.83	634.22	557.12-744.93	0.554
oxLDL (ng/mL)	447.10	280.39-687.64	428.05	339.93-766.24	0.820
ApoAI (ug/mL)	121.82	88.43-167.33	205.23	116.63-335.76	0.178
ApoAII (ug/mL)	161.92	109.35-183.76	215.52	158.76-257.10	0.178
ApoB100 (ug/mL)	323.13	226.70-411.68	784.69	311.30-1202.77	0.080
ApoCII (ug/mL)	10.32	10.02-12.84	20.11	13.06-23.54	0.007
ApoCIII (ug/mL)	26.33	17.18-40.17	70.97	34.72-82.22	0.029
ApoD (ug/mL)	14.10	9.20-22.51	15.18	6.27-23.04	0.820
ApoE4 (ug/mL)	0.00	0.00-0.00	0.00	0.00-306.56	0.554
ApoH (ug/mL)	0.00	0.00-7.75	3.33	0.42-29.06	0.249
ApoJ (ug/mL)	0.00	0.00-0.00	0.00	0.00-37.70	0.750
ApoM (ug/mL)	26.09	14.81-56.95	71.54	35.71-219.71	0.148
AOPP	0.00	0.00-0.00	2.28	0.00-5.96	0.211
T3 (pg/mL)	3.26	2.56-3.89	3.83	3.38-4.04	0.335
T4 (ug/dL)	4.69	4.23-5.77	3.96	3.49-4.46	0.022

AOPP: advanced oxidation protein products; Apo: apolipoprotein; IFNg: interferon-γ; IL: interleukin; IQR: interquartile range; oxLDL: oxidized low-density lipoprotein; T3: triiodothyronine; T4: thyroxine; TNF: tumor necrosis factor

Table S6. Postoperative laboratory parameters

Laboratory values	Median	IQR (25-75)
Sodium (mmol/L)		
PD 1	143.50	(140.25-144.75)
PD 2	141.50	(139.00-144.00)
PD 3	139.50	(138.00-143.00)
Potassium (mmol/L)		
PD 1	4.65	(3.87-5.00)
PD 2	4.95	(4.80-5.40)
PD 3	4.95	(4.70-5.47)
AST (UI/L)		
PD 1	53.50	(24.75-100.50)
PD 2	101.00	(72.50-121.00)
PD 3	109.50	(73.00-155.25)
ALT (UI/L)		
PD 1	26.00	(18.25-36.00)
PD 2	23.50	(20.75-39.00)
PD 3	31.00	(26.50-50.25)
GGT (UI/L)		
PD 1	50.00	(35.00-158.00)
PD 2	70.00	(27.00-114.00)
PD 3	64.50	(35.25-82.50)
ALP (UI/L)		
PD 1	60.00	(47.00-73.00)
PD 2	53.00	(41.50-88.25)
PD 3	59.00	(52.00-71.50)
LDH (UI/L)		
PD 1	427.50	(300.75-959.50)
PD 2	929.00	(789.00-1147.00)
PD 3	947.00	(771.00-1272.50)
Total Bilirubin (μmol/L)		
PD 1	22.70	(16.77-37.65)
PD 2	15.40	(13.90-25.38)
PD 3	15.00	(12.10-25.20)
Albumin (g/dl)		
PD 1	33.80	(31.40-46.40)
PD 2	32.30	(28.40-37.30)
PD 3	33.05	(27.03-36.13)
Total Protein (g/dl)		
PD 1	50.90	(44.95-53.55)
PD 2	51.75	(45.50-55.33)

ALP: alkaline phosphatase; ALT: alanine transaminase; AST: aspartate aminotransferase; GGT: gamma-glutamyl transpeptidase; LDH: lactate dehydrogenase; PD: postoperative day
